# The transcriptional characteristics of NADC34-like PRRSV in porcine alveolar macrophages

**DOI:** 10.3389/fmicb.2022.1022481

**Published:** 2022-10-19

**Authors:** Peixin Wang, Xin Ma, Riteng Zhang, Yongxin Zhao, Ruochen Hu, Chen Luo, Basit Zeshan, Zengqi Yang, Li Qiu, Juan Wang, Haijin Liu, Yefei Zhou, Xinglong Wang

**Affiliations:** ^1^College of Veterinary Medicine, Northwest A&F University, Yangling, Shaanxi, China; ^2^Faculty of Sustainable Agriculture, Universiti Malaysia Sabah, Sandakan, Sabah, Malaysia; ^3^Department of Life Science, Nanjing Xiaozhuang University, Nanjing, Jiangsu, China

**Keywords:** PRRSV, NADC34-like, comparative transcriptome, time-course transcriptome, WGCNA

## Abstract

The widespread and endemic circulation of porcine reproductive and respiratory syndrome virus (PRRSV) cause persistent financial losses to the swine industry worldwide. In 2017, NADC34-like PRRSV-2 emerged in northeastern China and spread rapidly. The dynamics analysis of immune perturbations associated with novel PRRSV lineage is still incomplete. This study performed a time-course transcriptome sequencing of NADC34-like PRRSV strain YC-2020-infected porcine alveolar macrophages (PAMs) and compared them with JXA1-infected PAMs. The results illustrated dramatic changes in the host’s differentially expressed genes (DEGs) presented at different timepoints after PRRSV infection, and the expression profile of YC-2020 group is distinct from that of JXA1 group. Functional enrichment analysis showed that the expression of many inflammatory cytokines was up-regulated following YC-2020 infection but at a significantly lower magnitude than JXA1 group, in line with the trends for most interferon-stimulated genes (ISGs) and their regulators. Meanwhile, numerous components of histocompatibility complex (MHC) class II and phagosome presented a stronger transcription suppression after the YC-2020 infection. All results imply that YC-2020 may induce milder inflammatory responses, weaker antiviral processes, and more severe disturbance of antigen processing and presentation compared with HP-PRRSV. Additionally, *LAPTM4A*, *GLMP*, and *LITAF,* which were selected from weighted gene co-expression network analysis (WGCNA), could significantly inhibit PRRSV proliferation. This study provides fundamental data for understanding the biological characteristics of NADC34-like PRRSV and new insights into PRRSV evolution and prevention.

## Introduction

Porcine reproductive and respiratory syndrome (PRRS) is an economically devastating pandemic of swine, which mainly causes respiratory failure, decreased boar semen quality, sow abortion, and stillbirth (Terpstra et al., 1991; Wensvoort et al., 1991; Benfield et al., 1992; Collins et al., 1992). The causative agent, porcine reproductive and respiratory virus (PRRSV), which belongs to the genus *Betaarterivirus* under the family *Arteriviridae* and can be divided into two major genotypes, PRRSV-1 (represented by Lelystad strain) and PRRSV-2 (represented by VR-2332 strain), according to The International Committee on Taxonomy of Viruses (ICTV) classification standards (Chen et al., 2006; Kuhn et al., 2016). PRRSV-2 was further classified into nine monophyletic lineages with several sub-lineages each (Shi et al., 2010); among them, the lineage 1.5 PRRSV-2 NADC34 became prevalent in the United States (in 2014), causing dramatic abortion storms in sow herds and high mortality among piglets (van Geelen et al., 2018). Recently, NADC34-like PRRSV has been detected in several Chinese provinces, including Liaoning, Henan, Fujian, and Jiangsu (Xu et al., 2022). These PRRSV-2 strains are also classified as lineage 1.5, with shared genomic features such as a 1–7-4 restriction fragment polymorphism (RFLP; *Mlu* I = 0, *Hinc* II = nt 88, 219, 360; *Sac* II = nt 24, 555) in the ORF5 sequence and a 100 amino acid continuum deletion in the NSP2 region compared to VR-2332 PRRSV (Bao and Li, 2021). Different NADC34-like PRRSV isolates exhibit distinct pathogenicity among themselves (Song et al., 2020; Yuan et al., 2022). Moreover, recombination events have been reported in NADC34-like PRRSVs in China (Xu et al., 2022); whether these phenomena will become the trigger of the next PRRS outbreak is still a concern.

Transcriptomics, an essential bioinformatics method in the post-genomic era, has been used widely in gene transcriptional structure identification, gene expression level quantification, and functional genomic investigation (Stark et al., 2019). It is also frequently applied in PRRSV infection assays to determine the gene expression changes in host cells under pathological conditions, thus further refining the virus-host interaction network (Miller et al., 2017; Sun et al., 2022). Research has focused on transcriptional characteristics under different organs/duration of infection and host/virus species. Several time-series studies showed that differentially expressed genes (DEGs) in PRRSV-infected PAMs were concentrated in innate immunity-related biological processes and pathways such as “pattern recognition receptors,” “cytokines,” and “antigen processing and presentation,” the level of enrichment showed dynamic changes throughout of the infection (Zeng et al., 2018). Moreover, an *in vivo* experiment showed that pigs could release characteristic signals of innate immunity in multiple tissues 3 days post-infection (dpi), with a host response summit at around 10 dpi (Lim et al., 2020). In addition, comparative transcriptome between three PRRSV strains showed that the host biological process stimulated by PRRSV infection was similar regardless of genotype differences. However, the response intensity triggered by PRRSV-2 was more prominent (Yang et al., 2018). The virulent PRRSV-1 strain, Lena, has a higher immune co-inhibitory receptor expression level than the avirulent 3294 strain (Sánchez-Carvajal et al., 2021). Besides, PRRSV-2 XJ17-5 induces innate immune-related genes such as cytokines and ISGs more strongly than the homologous JSTZ1712-12 (Li et al., 2021), which may indicate that the host immune system can respond more intensively to virulent PRRSV strains.

In the present study, host transcriptional characteristics of PAMs during NADC34-like PRRSV isolate YC-2020 infection were analyzed and compared with that of JXA1-infected PAMs. The results could provide essential data for the basic study of the emerging NADC34-like PRRSV and new insights into the prevention and control of PRRS.

## Materials and methods

### Cells and viruses

Porcine alveolar macrophages (PAMs) obtained from bronchoalveolar lavage of 12 4-week-old piglets (PRRSV-, PCV-, and PRV-negative) were cultured in Roswell Park Memorial Institute (RPMI) 1640 medium (Gibco, United States), while Monkey Embryonic Kidney Epithelial cells (MARC-145) were cultured in Dulbecco’s modified eagle medium (DMEM) both supplemented with 10% fetal bovine serum (FBS, Gibco), and 1% antibiotic/antimycotic solution (100 U/ml penicillin, 100 μg/ml streptomycin) in a humidified chamber at 37°C under 5% CO_2_ conditions; The PRRSV-2 strain JXA1 (GenBank No. EF112445) and YC-2020 (GenBank No. ON180781) propagated in PAMs were used in this study. The virus titer in PAMs was assessed through an indirect fluorescent antibody assay (IFA) conducted as previously described (Ma et al., 2021), with PRRSV-N mAb (monoclonal antibody, gifted by Professor Shuqi Xiao from NWAFU) and Goat Anti-Mouse IgG H&L (Alexa Fluor® 488) (Abcam, Cambridge, United Kingdom) as primary and secondary antibody, respectively. The 50% tissue culture infective dose (TCID50) was calculated using the Reed-Muench method.

### RNA preparation and sequencing

PAMs from every four piglets were equally mixed; the mixtures were then divided and designed as the negative control group (NC, without PRRSV infection) and two experimental groups [infected with one of PRRSVs described above at a multiplicity of infection (MOI) of 0.1]. All groups were incubated (NC with RPMI-1640) for 18, 28, and 38 h. All the samples were sent to Gene Denovo Biotechnology Co. (Guangzhou, China) for transcriptome sequencing (Supplementary Figure 1).

Total RNA was extracted using TRIzol reagent (Takara, Japan) following the manufacturer’s protocol. RNA quality was assessed on Agilent 2100 Bioanalyzer (Agilent Technologies, Palo Alto, CA, United States). mRNA with ployA tail was enriched by Oligo(dT) beads. Then the enriched mRNA was broken into short segments using fragmentation buffer and reverse-transcribed into cDNA using NEBNext Ultra RNA Library Prep Kit (NEB #7530, New England Biolabs, Ipswich, MA, United States). The purified double-stranded cDNA fragments were ligated to Illumina sequencing adapters after end repair & A base adding. Ligated fragments were further subjected to size selection by agarose gel electrophoresis and polymerase chain reaction (PCR) amplification. The resulting cDNA library was sequenced using Illumina NovaSeq 6000, and 150 bp paired-end reads were generated. All raw data generated in this study were deposited in the NCBI Sequence Read Archive (SRA) database under the accession number PRJNA857481.

### Data processing and DEGs analysis

High-quality and adapter-trimming reads were filtered by Fastp (v0.22.0) (Chen et al., 2018) from the raw data (FASTQ format) and then aligned to the ribosomal RNA (rRNA) database of Sus scrofa by Bowtie2 (v2.4.4) to remove rRNA-derived reads. HISAT2 aligner (version 2.2.1) (Kim et al., 2015) was used for mapping clean reads to the Ensembl Sscrofa11.1 reference genome (release 104). Gene-wise abundances of each sample were calculated based on the exons annotated in the Ensembl Sscrofa11.1 GTF file (release 104), using the FeatureCounts software (v2.0.1) (Liao et al., 2014). Moreover, the fragments per kilobase per million mapped reads (FPKM) measurement for each gene in each sample was also calculated as auxiliary data for downstream analysis.

Differentially expressed genes analyses were performed with R package DESeq2 (v1.34.0) (Love et al., 2014). To reduce statistical bias caused by compositional and size differences between the libraries, we conducted the raw counts’ normalization using the Relative Log Expression (RLE) method first. Then estimated the log2 fold change (LFC) of gene expression between each comparison group based on the above-mentioned experimental variables [treatment, hours post infection (hpi)] and “shrank” them through *apeglm* and *ashr* algorithms. The false discovery rate (FDR) was applied for multiple testing corrections of raw *p-*values (using the *Benjamini–Hochberg* (BH) method). A |LFC| > 1, an FDR-adjusted *p*-value (padj) < 0.05 and FPKM subtraction >2 were set as the threshold for determining statistically significant DEGs.

### Functional annotation analysis

To identify relevant biological processes and pathways involved in each comparison group, functional gene set enrichment analysis (GSEA) of gene ontology (GO) and Kyoto encyclopedia of genes and genomes (KEGG) pathway annotations were conducted with the R package ClusterProfiler (v4.2.1) (Wu et al., 2021). Signal-to-noise ratio (SNR) was selected as the gene-ranking method, and the result of gene set enrichment with a |normalized enrichment score (NES)| > = 1.5 and *Q* value <0.1 was considered to be statistically significant. Enriched GO terms were visualized in Cytoscape (v3.9.0) (Shannon et al., 2003). The EnrichmentMap plugin (Merico et al., 2010) was used to construct a similarity-based network, in which GO terms were represented as nodes and edges were drawn based on a combined similarity coefficient (Jaccard + Overlap score > 0.4). The titles of term clusters were annotated by the AutoAnnotate plugin (Kucera et al., 2016), using the Markov cluster algorithm (MCL).

### Construction of weighted gene co-expression network

To explore the potential relationship between genes more comprehensively from the perspective of overall expression trend, we constructed the mRNA co-expression network by using the R package WGCNA (v1.7) (Langfelder and Horvath, 2008) with entire samples. The specific process can be roughly divided into the following steps: (1) import gene expression data from all samples and obtain a similarity matrix from genes’ correlations calculated by the Pearson method; (2) select an optimal soft thresholding power that can bring the target network closer to a scale-free network (scale-free fit R^2 > 0.9), and transform the similarity matrix into an adjacency matrix; (3) furthermore, transform the adjacency matrix into a topological overlap matrix (TOM) and perform gene clustering on TOM-based dissimilarity matrix (dissTOM); (4) utilize the dynamic tree cut algorithm to determine the co-expression gene sets, namely modules; (5) the module eigengene (ME) of each module is calculated, based on which the correlations between all modules are obtained, and then modules with a dissimilarity <0.2 are merged into a new one.

### Identification of key modules and functional enrichment analysis

The Pearson correlation coefficients between the MEs of each module and each sample trait were calculated to estimate the module-trait associations, and relationships with a *t*-test *p-*value <0.01 were considered statistically significant. Meanwhile, to explore the biological processes in which members within the module may be involved, we performed GO and KEGG functional enrichment analyses for each module using the R package ClusterProfiler (v4.2.1) with the over-representation analysis (ORA) method. Gene sets were significantly over-represented with an adjusted *p-*value less than 0.05.

### Identification and functional validation of hub genes

Hub genes exhibit high interconnection with other genes in a module and are generally considered to play important roles in a scale-free gene expression network. Intra-modular Connectivity function within the WGCNA R package was used to calculate gene connectivity, and the hub candidates were recognized as the top 5% of genes with the highest connectivity in each module. In addition, we also determine module membership (MM, measurement of gene-module correlation) and gene significance (GS, measurement representing the correlation between the gene and a given trait) for each gene to assist the identification process.

The full-length of selected hub genes’ coding sequences (CDSs) were amplified from the RNA extraction of infected PAMs and cloned into the vector pCDNA-KHA (Miaoling biology, Hubei, China) to generate three recombinant plasmids pCDNA-LITAF, pCDNA-LAPTM4A, and pCDNA-GLMP, respectively. MARC-145 cells transfected with specific plasmid were then infected with PRRSV strain JXA1, cell lysates were collected for RT-qPCR assay and Western blot assay at both 24- and 48 hpi.

### RT-qPCR and Western Blot

Total extracted RNA was reversely transcribed to cDNA using M-MLV Reverse Transcriptase (Genstar, China). RT-qPCR was carried out with a real-time thermocycler (Four-channel, Tianlong, China) using the 2× Fast qPCR Master Mixture (DiNing, Beijing, China), following the instruction manual. All reactions were performed in triplicate. The previously described method of 2^−ΔΔCT^ was used to calculate the relative expression level of target mRNAs (Livak and Schmittgen, 2001). The β-actin gene was used to normalize the fold changes in expression. Supplementary Table 1 shows the respective primer sequences of the reference gene (β-actin) and selected mRNA transcripts.

Cell lysates were denatured in 1 × protein loading buffer (10 mM Tris–HCl, pH 8.5, 50 mM DTT, 1% SDS, 10% glycerol, and 0.008% bromophenol blue) by heating for 5 min at 100°C. Protein samples in cell lysates were separated by SDS-PAGE and transferred to nitrocellulose membranes (Millipore, United States) as described previously (Lv et al., 2018). Membranes were then blocked with 5% skimmed milk in TBST (YaMei, Shanghai, China), followed by incubation with the primary antibody (PRRSV-N mAb, SLA-DRA mAb (gifted by Professor Qin Zhao from NWAFU), β-actin (Sungene Biotech, Tianjin, China), HA (Sungene)) and secondary antibody (HRP-labelled goat anti-mouse IgG (Sangon Biotech, Shanghai, China)), both diluted in TBST containing 2% BSA. The resulting signals were visualized by ECL ENhanced Kit (DiNing) with an Odyssey infrared imaging system (LI-COR, Nebraska, United States), and the protein band intensity was measured using ImageJ quantification software (available online at: https://imagej.net). All washes were using TBST, six times every 6 min.

## Results

### NADC34-like PRRSV YC-2020 has more viral transcripts

After trimming, filtering & rRNA removal, 1,136 million 150 bp pair-end high-quality reads were obtained from all 27 objects. Each sample was mapped to the pig reference genome, the YC-2020 genome, and the JXA1 genome, respectively, with an overall reads mapping rate of 96% or more (Supplementary Table 2). No virus reads mapping occurred in the NC group. No cross-infection was noticed between JXA1 and YC-2020 groups as only corresponding virus reads were detected, indicating that all the data are suitable for further analysis.

**Figure 1:**
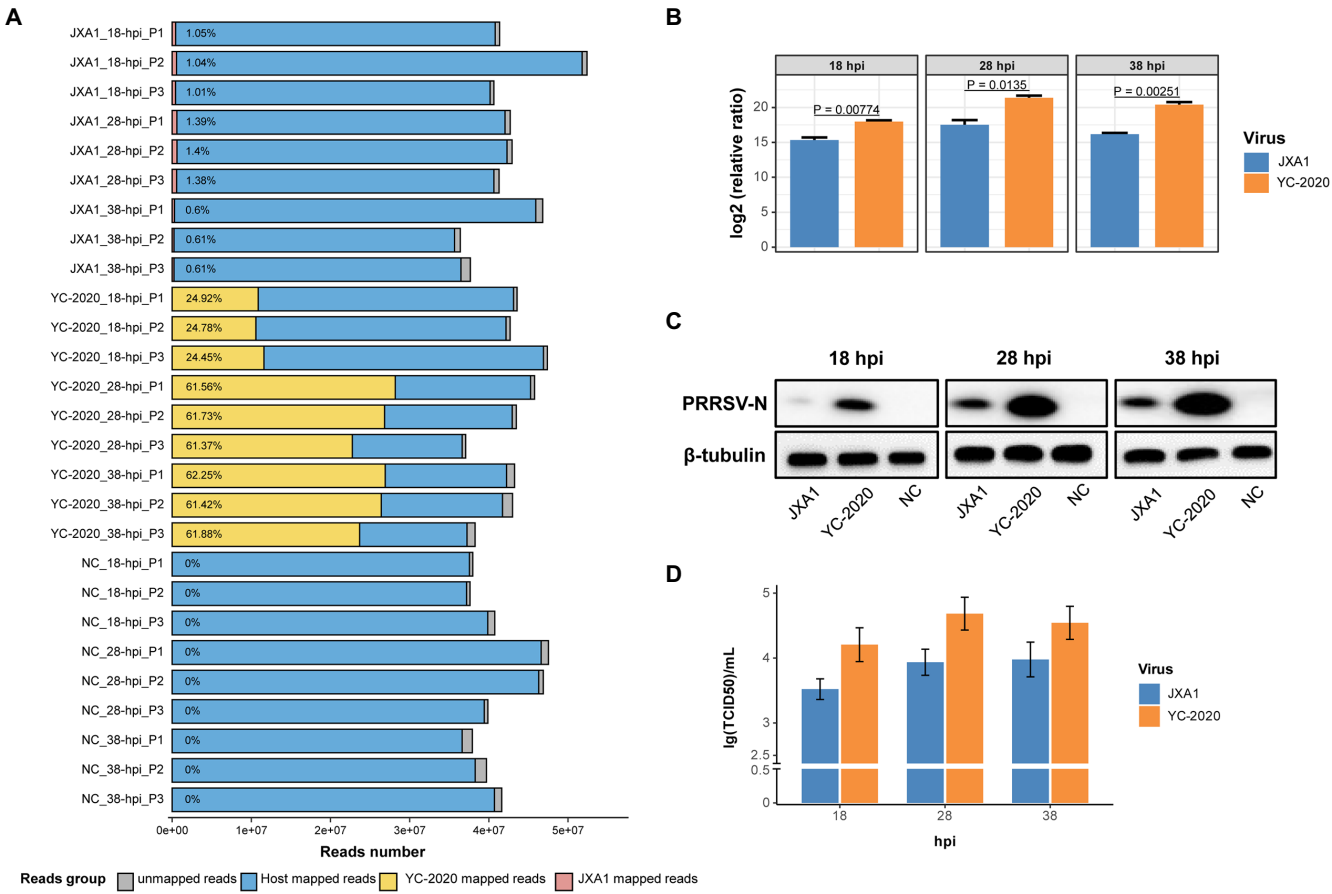
RNA-seq mapping statistic and virus transcripts validation assays. **(A)** Bar plot of RNA-seq mapping statistic. Y-axis represents 27 samples that were generated by treatment (YC-2020, JXA1, and NC), time (18-, 28-, and 38-hpi), and three replicates (P1, P2, and P3); numbers within the bars represent the percentage of virus-derived reads in each sample. **(B)** The viral loads of two PRRSV strains at different hpi were validated by RT-qPCR. Data are mean ± SD from three independent experiments, and *p-*values are calculated by unpaired two-tailed Student’s *t*-test. **(C)** Detection of PRRSV-N protein expression at different hpi by Western Blot assay. **(D)** Determination of virus titer at different hpi by TCID50 assay.

Notably, the YC-2020 PRRSV-derived reads reached a quarter of the total sequencing data at 18 hpi and rose above 60% at 28 hpi, while JXA1-derived reads hovered around 1% throughout the observation (Figure 1A). This phenomenon was further confirmed at the transcription, protein, and virus titer levels by RT-qPCR (Figure 1B), Western Blot (Figure 1C), and TCID50 (Figure 1D) assays, respectively.

### Gene expression profiles in NADC34-like PRRSV-infected PAMs

**Figure 2:**
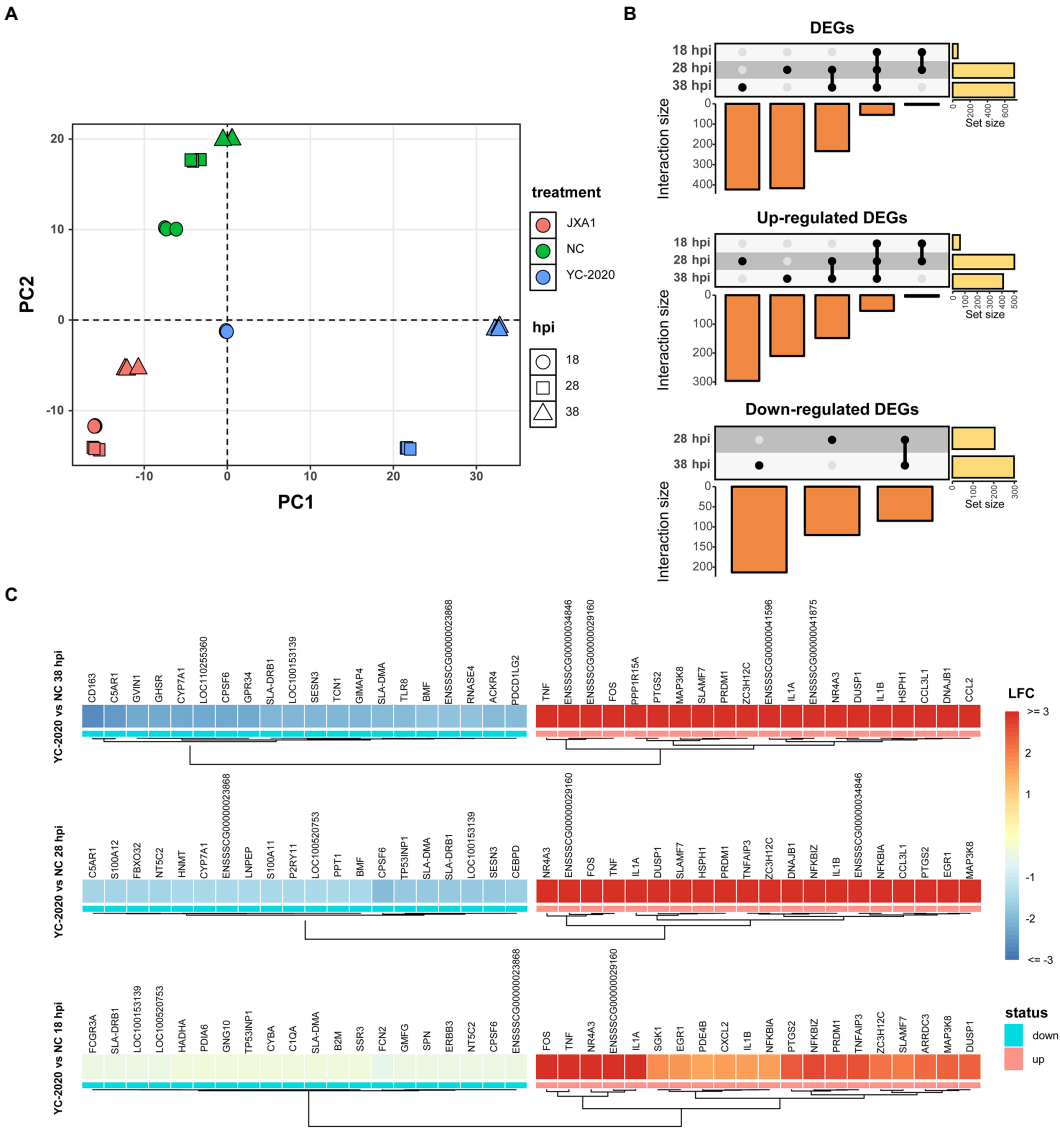
DEGs analysis between YC-2020 and NC group. **(A)** Principal component analysis of 27 samples based on gene expression. The eigenvalues from the first two principal components (PC1 and PC2) are plotted. The characteristics of each sample are marked by both color and shape aesthetics. **(B)** UpSet plot shows the dynamic distribution of DEGs under each comparison. Each vertical bar represents the number of DEGs in one distribution set, and the horizontal bars represent DEGs’ total number at different hpi. **(C)** Heat map of the expression of the top 20 up- and down-regulated DEGs (sorted by LFCs) at three timepoints.

The principal component analysis (PCA) (Figure 2A) showed no significant segregation of replicates; moreover, the first principal component explained the differences in host gene expression caused by virus type, suggesting that YC-2020 may have different infection characteristics from JXA1. Time-course DEGs analysis based on the YC-2020 and NC groups revealed dynamic changes in gene expression. DEGs with the threshold described in section “Methods” were identified as 59 at 18 hpi but significantly increased to 708 at 28 hpi and maintained the trend at 38 hpi. Up-regulated DEGs accounted for a more significant proportion (Figure 2B; Supplementary Table 3). The top 20 up-and down-regulated genes (sorted by LFCs) at each hpi were listed in Figure 2C, where the presence of *TNF*, *IL-1A*, *IL-1B*, *CCL2*, *SLA-DRB1*, *SLA-DMA*, and *GVIN1* suggested that the DEGs were possibly involved in the inflammatory response, antigen presentation, and antiviral process.

**Figure 3:**
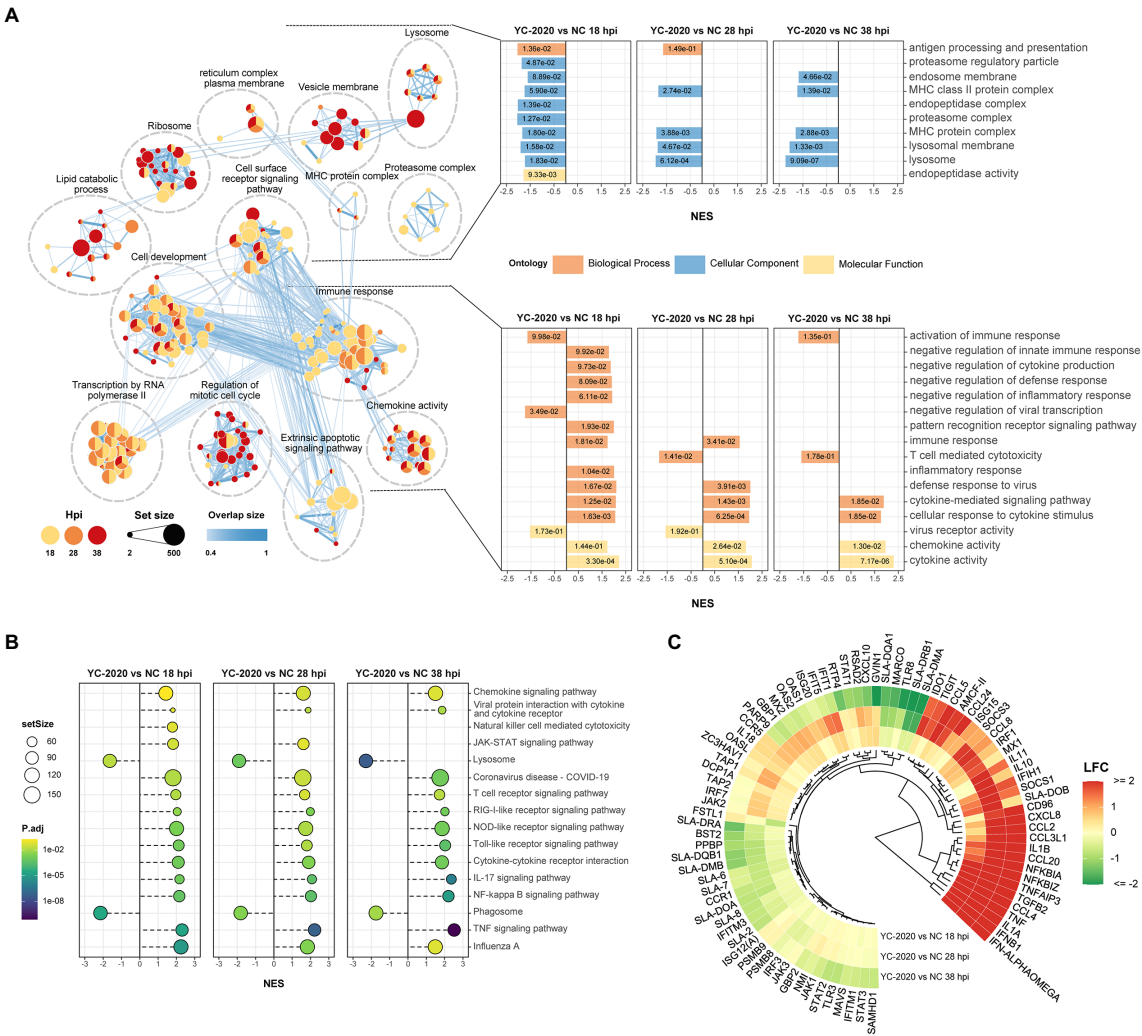
Functional annotation analysis of DEGs determined from the YC-2020 and NC group. **(A)** GO enrichment at different hpi. The left side of the panel shows the significantly enriched GO terms (|NES| ≥ 1.5, padj <0.1) obtained at all three hpi. Gene sets are represented by nodes, which color corresponds to hpi; node size corresponds to the number of gene members within them; the size and color of the edges correspond to the similarity score between terms. All GO terms are clustered according to their gene members’ overlap size, and each cluster’s subject is written on top. GO terms associated with immune response, antigen processing and presentation are shown in the panel’s lower and upper bar plots on the right side. Each bar contains a padj of the permutation test. **(B)** Lollipop plot of the significantly enriched KEGG pathway at different hpi. **(C)** Heat map of the expression profiles of hotspot genes associated with innate immunity. Each layer of circles represents a comparison group, and the selected genes include common cytokines, ISGs, MHCs, and critical regulators in associated pathways.

Gene set enrichment analysis based on GO and KEGG databases indicated that many biological processes and gene pathways were significantly enriched during YC-2020 PRRSV infection. The immune-related GO terms, such as “immune response,” “defense response to virus,” “chemokine-mediated signaling pathway” were positively enriched (NES > 0); interestingly, most of these enriched states further pointed to the negative regulation (e.g., “negative regulation of innate immune response,” “negative regulation of inflammatory response,” “negative regulation of defense response,” “negative regulation of cytokine production”); a few genes sets that presented as a negative enrichment state (NES < 0) were also focused on associative processes adverse to viral invasion (e.g., “activation of immune response,” “negative regulation of viral transcription”). The enrichment number of general immune-related terms diminished along with the duration of infection. Meanwhile, GO terms related to antigen processing and presentation, such as “antigen presentation and processing,” “MHC protein complex,” “lysosome,” and “proteasome” were in a negative enrichment state at all three timepoints (Figure 3A). Results of KEGG pathways were similar to that of GO terms (Figure 3B). Furthermore, the expression profiles of innate immune-related marker genes also elucidated that the majority of cytokines, including type I interferons (IFNs, *IFN-ALPHAOMEGA,* and *IFNB1*), inflammatory chemokines (*CCL4, CCL2, CCL3L1, CCL5, CCL20, CXCL8,* and *AMCF-II*), interleukin (IL) family (*IL-1A, IL-1B,* and *IL-11*) and NF-κB pathway inhibitory proteins (*NFKBIA, NFKBIZ,* and *TNFAIP3*) showed significantly high expression level under all observation points. In contrast, the expression of some inflammation-inducing factors (*PPBP, MARCO*) was consistently down-regulated in the middle and late stages of infection. Most interferon-stimulated genes (ISGs, such as *GVIN1, RTP4, IFIH1, IFIT1, IFIT5, MX1, MX2, RSAD2,* and *BST2*) exhibited an increase in transcript amount at 18 and 28 hpi but a decrease at 38 hpi. The expression level of MHC class II molecules associated with antigen presentation (*SLA-DMA1, SLA-DQB1, SLA-DMB, SLA-DRB1, SLA-DQA1,* and *SLA-DRA1*) was down-regulated at all observed timepoints (Figure 3C; Supplementary Table 4).

### NADC34-like PRRSV YC-2020 infection induced more significant gene transcriptional regulation

**Figure 4:**
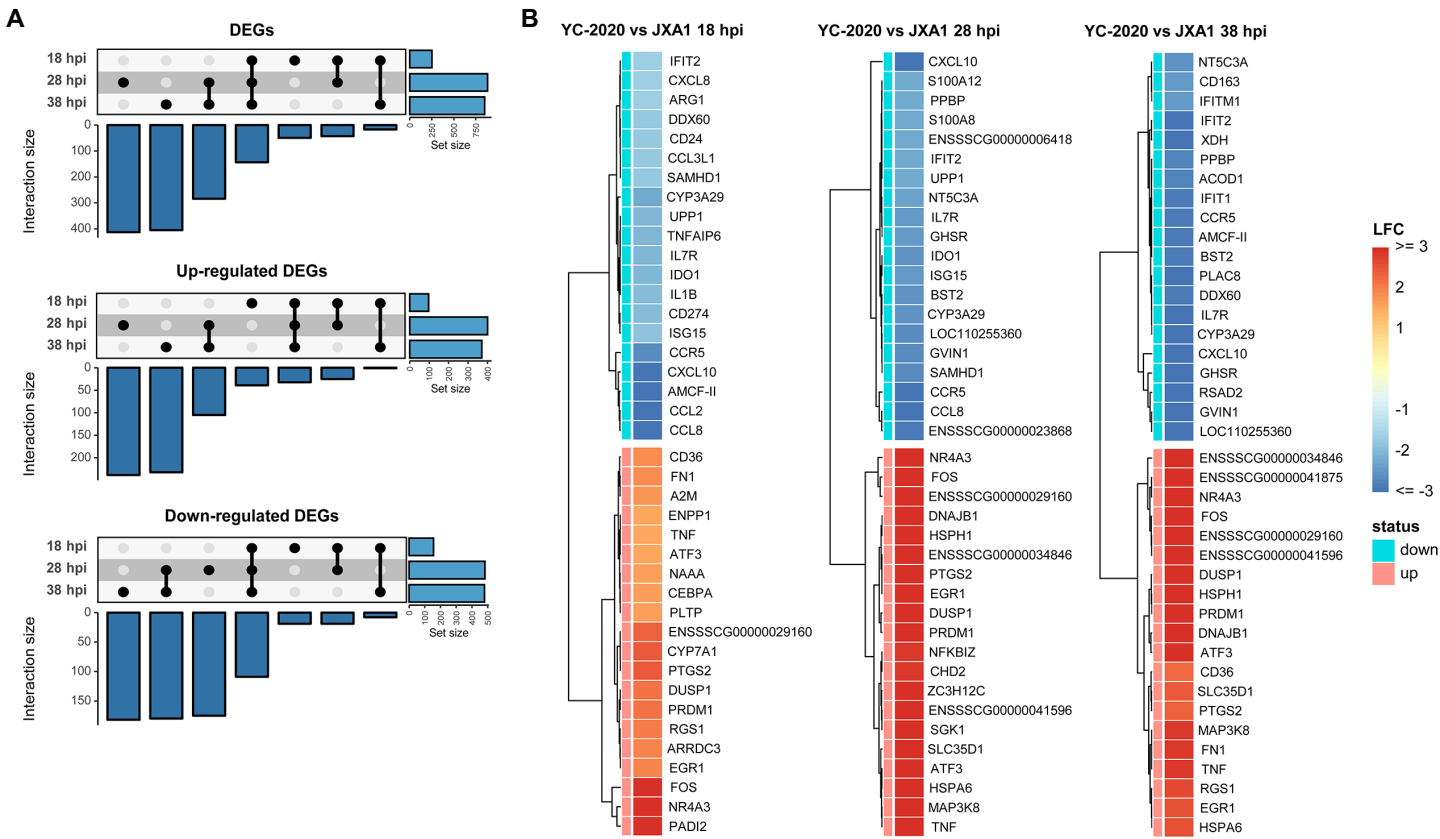
DEGs analysis between YC-2020 and JXA1 group. **(A)** UpSet plot shows the dynamic distribution of DEGs under each comparison. **(B)** Heat map of the expression of the top 20 up- and down-regulated DEGs (sorted by LFCs) at three timepoints.

**Figure 5:**
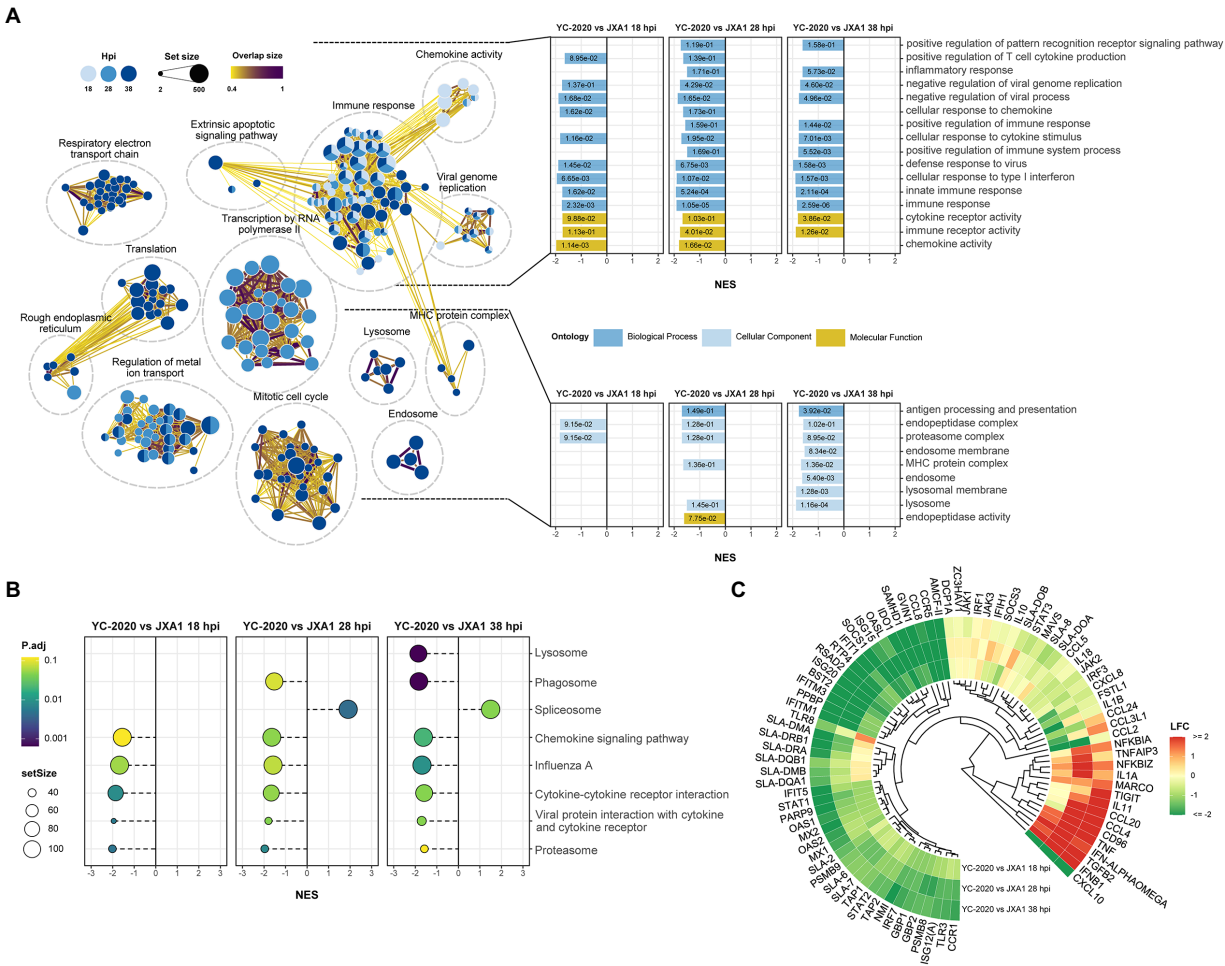
Functional annotation analysis of DEGs determined from the YC-2020 and JXA1 group. **(A)** GO enrichment at different timepoints post-infection. The left side of the panel shows the significantly enriched GO terms (|NES| ≥ 1.5, padj <0.1) obtained at all three timepoints. Gene sets are represented by nodes, in which color corresponds to hpi; node size corresponds to the number of gene members within them; the size and color of the edges correspond to the similarity score between terms. All GO terms are clustered according to their gene members’ overlap size, and each cluster’s subject is written on top. GO terms associated with immune response, antigen processing and presentation are shown in the panel’s upper and lower bar plots on the right side. Each bar contains a padj of the permutation test. **(B)** Lollipop plot of the significantly enriched KEGG pathway at different hpi. **(C)** Heat map of the expression profiles of hotspot genes associated with innate immunity. Each layer of circles represents a comparison group, and the selected genes include common cytokines, ISGs, MHCs, and critical regulators in associated pathways.

DEGs analysis between YC-2020 and JXA1 groups was performed to explore the distinctions in transcriptional characteristics of NADC34-like- and HP-PRRSV-infected PAMs. Similar to YC-2020 vs. NC group, DEGs between YC-2020 and JXA1 group also displayed a considerable increase at 28 hpi and did not overlap well with those at 38 hpi (Figure 4A; Supplementary Table 3). The significantly up-regulated genes (such as *DNAJB1*, *FOS*, *ATF3*, and *NR4A3*) were focused on the transcriptional regulation process, whereas the significantly down-regulated genes (*CXCL10, CCL2*, *CCL8*, *AMCF-II*, *BST2, RSAD2,* and *GVIN1*) were annotated to the immune response (Figure 4B). GO terms and KEGG pathways involved in the viral immune response and its positive regulation (including “positive regulation of pattern recognition receptors,” “positive regulation of immune response,” “Cytokine-cytokine receptor interaction,” “influenza A”) were found to be negatively enriched throughout the observation phase. Negative enrichment terms related to the negative regulation of viral replication (“negative regulation of viral process,” “negative regulation of viral genome replication”) also suggested that PAMs infected with YC-2020 PRRSV exhibited weaker restraint against the virus (Figures 5A,B). Part of ILs (*IL-1A*, *IL-11*) and chemokines (*CCL4*, *CCL20*) showed higher expression in the YC-2020 group instead of JXA1 group. However, toll-like receptors (TLRs, *TLR3,* and *TLR8*), interferon regulator factors (IRFs, *IRF3,* and *IRF7*), most of the cytokines (*CXCL10, CCR5, CCL2, CCL8, AMCF-II*), ISGs (such as *ISG15, ISG20, GBP1, GVIN1, RTP4, NMI, PARP9, IFIT1, IFITM3, OAS1, RSAD2,* and *BST2*), antigen processing and presentation associated genes (including *SLA-DMA1, SLA-DRB1, TAP1, TAP2, PSMB8, PSMB9*) presented lower transcription levels in the comparison of the YC-2020 and JXA1 groups, and fold change of the above genes expanded along with the infection duration (Figure 5C; Supplementary Table 4). The results of DEGs and functional enrichment analysis between JXA1 and NC groups were presented in Supplementary Figures 2, 3 to understand the transcriptional differences between the two strains more sufficiently.

**Figure 6:**
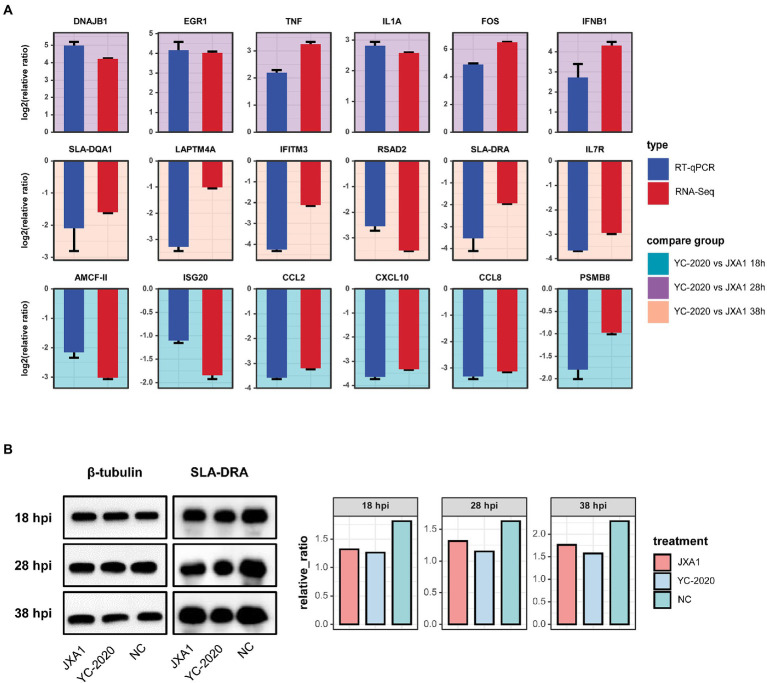
Validation of RNA-Seq data. **(A)** Comparative bar plot between RT-qPCR validation of the 18 selected DEGs and their RNA-seq data. Each subplot corresponds to a gene, and the background color represents the comparison group. All results are shown as means ± SE. **(B)** Detection of SLA-DRA protein expression at different hpi by Western Blot assay. Tubulin was used as an internal reference. **(C)** Proteins were quantified with gray intensity analysis software.

Eighteen DEGs selected from different comparison groups were validated by RT-qPCR. Despite slight variations in the magnitude of changes in a few genes, the RT-qPCR results exhibited superior consistency with RNA-Seq, indicating the sequencing data’s reliability (Figure 6A). *SLA-DRA* was chosen as the representation protein to confirm that YC-2020 PRRSV possessed a stronger inhibitory capacity for antigen presentation than JXA1 PRRSV through the Western Blotting assay. As the gray intensity analysis showed, the expression level of *SLA-DRA* was lower in both virus groups than that in the NC group at all observation points, and YC-2020 showed a more significant down-regulation trend than JXA1 from 28 hpi (Figure 6B).

### The identification of key modules and hub genes through WGCNA

**Figure 7:**
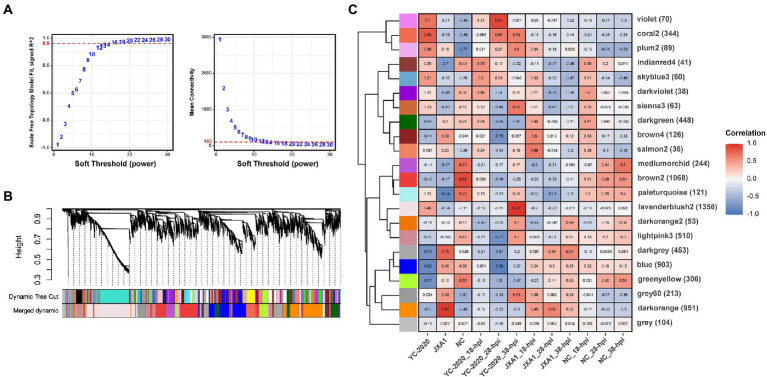
Gene co-expression network construction and module identification. **(A)** Determination of soft-thresholding power in WGCNA. The numbers in the panel are the power candidates. **(B)** Hierarchical clustering dendrograms of identified DEGs. Modules correspond to branches and are labeled by colors as indicated by the first color band underneath. After the ME-based hierarchical clustering, the original modules are merged and presented in the second color band. **(C)** Heatmap of module-trait associations. Correlation is written into each cell with fill color as an indicator.

To discover some novel possible intergenic linkages from the overall expression trends, a gene co-expression network was constructed by WGCNA. The hierarchical clustering results obtained using the gene FPKM values in each sample detected no significant outlier. 14 was set to be the optimal power to construct a scale-free, unsigned gene expression network after multiple testing (Figure 7A). Afterward, the expression profiles of 7,597 genes were converted into a TOM-based gene-wise clustering tree, and 22 modules were finally identified (Figure 7B; Supplementary Table 5).

**Figure 8:**
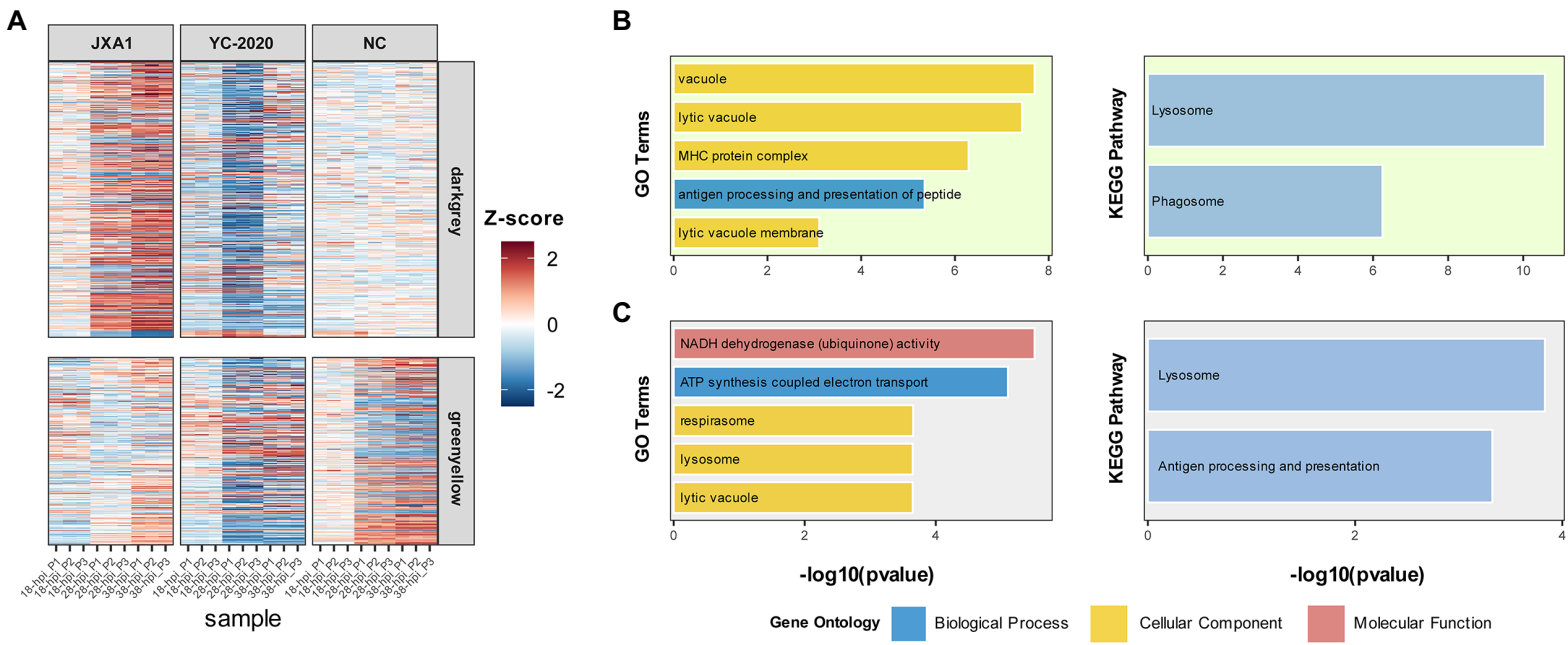
Information on modules of interest. **(A)** The expression profile of gene members in the darkgray and greenyellow modules in all samples. Each row corresponds to a gene, while each column corresponds to a sample. FPKM of each gene across samples is centralized and normalized for more precise visualization. GO and KEGG-based functional enrichment analysis of greenyellow and darkgrey modules are presented in **(B,C)**, respectively.

**Figure 9:**
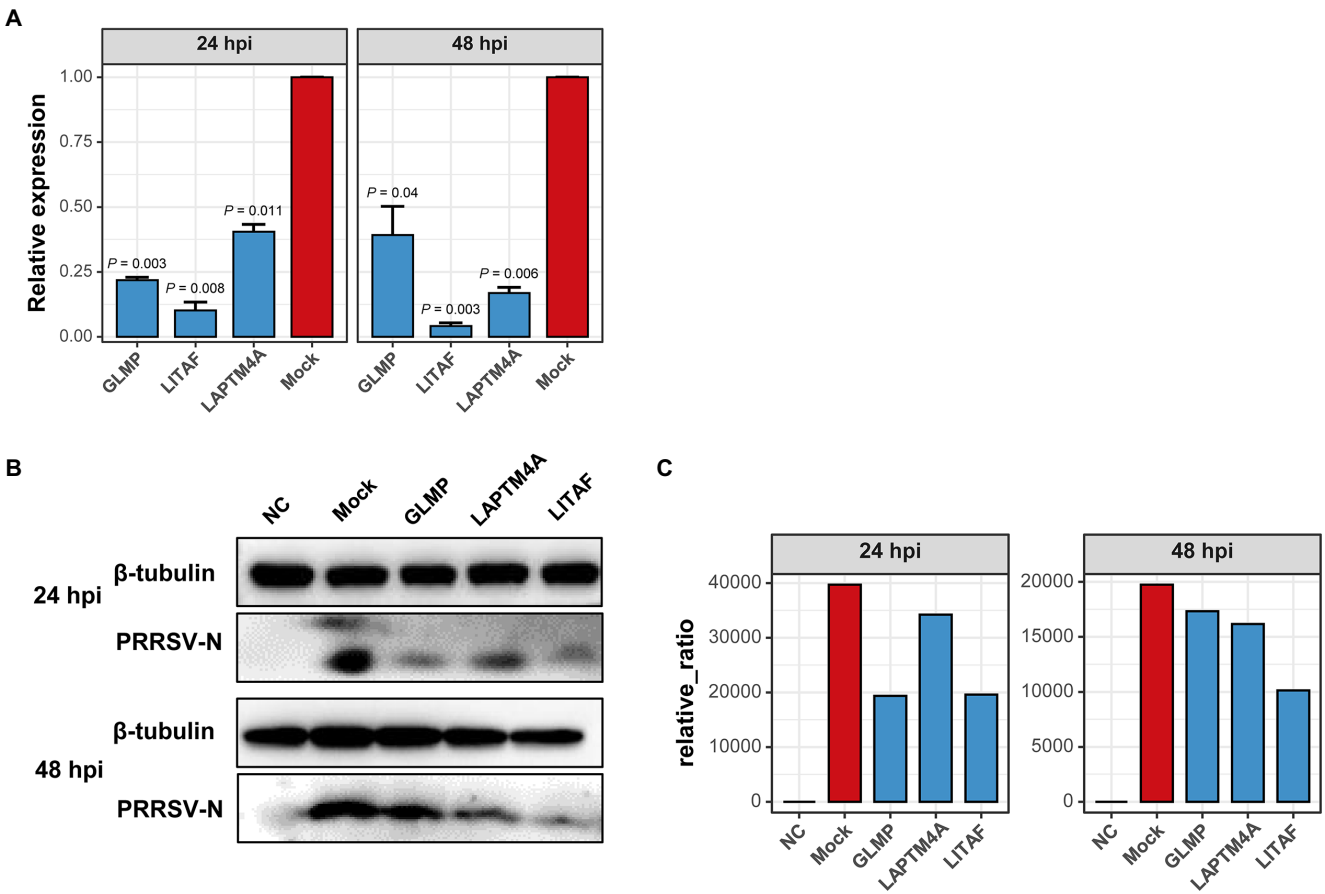
Over-expression of three hub-genes down-regulates PRRSV replication *in vitro*, respectively. **(A)** PRRSV proliferation was quantified by detecting mRNA transcription of protein N in Marc-145 cells overexpressing hub genes or not. Overexpressing hub genes downregulated virus-derived N expression significantly at both 24- and 48 hpi relative to that in mock cells. Data are mean ± SD from three independent experiments, and *p-*values are calculated by unpaired one-tailed Student’s *t*-test. **(B)** PRRSV-derived N expression decreased significantly in cells transfected with hub genes detected by Western Blot using anti-PRRSV-N pAb. Tubulin was used as an internal reference. **(C)** Proteins were quantified with gray intensity analysis software.

The relationship between each module and sample information (Supplementary Table 6), and biological processes involved in modules, were determined by correlation calculations and ORA-based functional enrichment analysis before the identification, respectively. The greenyellow and darkgrey were considered key modules because both of them contained terms or pathways associated with antigen processing and presentation (Figures 8B,C); meanwhile, these two modules were highly negatively correlated with the “YC-2020 PRRSV infection” trait (r^greenyellow^ = −0.77, *P*^greenyellow^ = 2.73e-06, r^darkgrey^ = −0.75, *P*^darkgrey^ = 7.45e-06, Figure 8C). The overall expression profiles of all genes in key modules are shown in Figure 8A. Based on the threshold (top 5% of kIM) and auxiliary selection criteria (|MM| > 0.85, |GS| > 0.4, mean expression counts >4,000, and max |LFCs| > 1), *LAPTM4A*, *GLMP*, *LITAF* in key modules were finally selected as hub genes for further study (Supplementary Table 7). MARC-145 cells were transfected with pCDNA-LITAF, pCDNA-LAPTM4A, and pCDNA-GLMP, respectively. And then infected with JXA1 at 12 h post transfection. Cell lysates were collected at 24 h and 48 h after virus infection, followed with RT-qPCR and Western Blot. Three hub genes could significantly inhibit PRRSV propagation at mRNA-expression and protein levels compared to mock; among them, *LITAF* showed a stronger inhibitory effect (Figure 9).

## Discussion

Porcine reproductive and respiratory syndrome virus infection triggers a series of cellular responses in PAMs, forming a complex viral-host gene expression regulation network. Considering the differences in pathogenicity caused by rapidly recombinant events in PRRSV and the limitation of traditional molecular biology techniques, high-throughput transcriptome sequencing was conducted to analyze the host transcriptional characteristics of NADC34-like PRRSV YC-2020 infection to obtain important data on the organismal response induced by this emerging strain. At the same time, a comparative analysis with JXA1 (one of the representative strains of HP-PRRSV, often compared with other novel strains) was also carried out to explore the host transcriptional differences between NADC34-like and highly pathogenic strains infection. The results of each specific comparison group demonstrated a substantial increase in the number of DEGs at 28 h after YC-2020 infection and a large proportion of up-regulated genes, implying that the response of PAMs was significantly activated between 18 and 28 h. From the comparison between the YC-2020 and JXA1 group, down-regulated DEGs at 28 and 38 h showed a relatively high overlap degree, suggesting that PAMs infected with NADC34-like PRRSV may possess sustained suppressive effects in some aspects relative to those infected with HP-PRRSV.

In this study, functional enrichment analysis was performed by GSEA instead of ORA method to reveal the pathways annotated by differential genes since the former can show the enrichment status of the target gene set in the whole gene list. More importantly, GSEA can effectively avoid the information loss caused by the hard threshold. The classified enrichment results are mainly related to immune response, lipid metabolism, cell cycle, translation, and other processes. Immune response was chosen as the key focus of this study. Innate immunity, the first line of host defense to limit virus transmission and regulate acquired immunity, activates a series of related signaling pathways in cells with pattern recognition receptor binding as a trigger (Zeng et al., 2018). The gene sets “immune response,” “defense response to the virus,” “pattern recognition receptor signaling pathway” were positively enriched after YC-2020 infection, but negatively enriched in comparison with JXA1; positive enrichment was also observed in the anti-immune regulatory gene sets at 18 hpi, indicating that the activation and suppression of innate immunity coexisted after the YC-2020 infection, however, the activation level was lower than that of JXA1.

Inflammation is one of the essential features of the immune response, and it can be induced after PRRSV infection, which promotes immune cell infiltration (Xiao et al., 2010). Various inflammatory cytokines such as TNF (*TNF*), some ILs (*IL1α*, *IL1β*), and chemokines (including *CCL2*, *CCL4*, *CCL5*, *CXCL8*) were significantly up-regulated under all observation points after YC-2020 infection. Meanwhile, anti-inflammatory factors such as *TNFAIP3, NFKBIA, NFKBIZ, SOCS1, SOCS3,* and *IL-10* were elevated, with the reverse trends for the inflammation-inducing genes *PPBP* and *MARCO* at 38 hpi, suggesting that the pro- and anti-inflammatory responses might coexist in PAMs during YC-2020 infection. These phenomena were also captured in a recent study (Chaudhari et al., 2021), where the author thought that the production of pro-inflammatory factors originates from the body’s immune defenses, while anti-inflammatory factors are attributed to PRRSV-induced negative regulation of immunity. The majority of inflammatory cytokines presented lower expression levels except for *TNF*, *IL1α*, and some chemokines (*CCL4, CCL20*) in the YC-2020 vs. JXA1 groups, but expression of the anti-inflammatory gene markers (*NFKBIA, NFKBIZ,* and *TNFAIP3*) was higher, indicating that YC-2020 PRRSV may cause a weaker inflammatory response than HP-PRRSV through stronger inhibition of the NF-κB signaling pathway (Oeckinghaus and Ghosh, 2009; Das et al., 2018). Previous studies have demonstrated a similar theory both *in vitro* and *vivo*, where HP-PRRSV tended to generate more severe lung damage by leading to excessive inflammation but could be eliminated quickly from the organism (Weesendorp et al., 2014). Therefore, we consider that a milder inflammatory response is also a strategy for YC-2020 to evade the host’s immune response.

As a critical pathway in the anti-PRRSV process, cytokines, especially the IFN-mediated JAK–STAT pathway, have been extensively studied (Huang et al., 2015). IFN-I activated by several transcription factors such as NF-κB and IRFs triggers the production of numerous ISGs through this pathway, yet PRRSV can counteract it through viral proteins (Schoggins and Rice, 2011; Wang et al., 2021). In the present study, both *IFN-ALPHAOMEGA* and *IFNB1* were extremely significantly up-regulated (LFCs >5) at three timepoints after YC-2020 infection, which has been detected in some previous RNA-Seqs toward PRRSV-2 infection (Lim et al., 2020; Chaudhari et al., 2021). Intriguingly, most of the downstream ISGs’ up-regulation levels were much lower in the comparison between YC-2020 and NC, with some even exhibiting a reversal at 38 hpi. Except for *DCP1A* and *ZAP* (*ZC3HAV1*), most ISGs in the YC-2020 group showed a significantly lower expression than the JXA1 group, and gaps widened with the continuance of infection, which imply that YC-2020 may possess a more powerful capacity to weaken the host’s antiviral responses. Considering the remarkably lower transcription of *STAT1* and *STAT2* was also founded in the comparison between YC-2020 and JXA1 groups; whether viruses would primarily rely on transcriptional repression of these two genes to limit the production of ISGs under the circumstance of highly expressed IFNs remains to be explored in depth.

Recently, antigen processing and presentation has become a hot spot because of its role as a “bridge” between innate and adaptive immunity. The impairment of this process is considered one of the main tactics of PRRSV-induced immunosuppression (Chaudhuri et al., 2016; Liang et al., 2017; Zeng et al., 2018). GO and KEGG terms such as “antigen processing and presentation,” “lysosome” and “proteasome” were enriched negatively both in the comparison of YC-2020 vs. NC and YC-2020 vs. JXA1 groups throughout the experiment. Besides, the transporter associated with antigen processing (TAP, *TAP1, TAP2*) and most MHCs showed a decrease in expression at 28, 38 h post-YC-2020 infection, with a stronger downtrend than the JXA1 group. The results indicate that YC-2020 may cause a more severe antigen-presentation disturbance than HP-PRRSV by inhibiting the transcription of genes associated with phagosome and MHC complexes.

We also discovered that the YC-2020-derived viral reads accounted for a significantly higher percentage than JXA1, once reaching more than 60% of the belonging sample’s total data volume. The proportion of viral-derived reads in existing PRRSV-associated transcriptome studies typically ranges from 1 to 20% (Liang et al., 2017; Wu et al., 2020; Chaudhari et al., 2021); however, a recent analysis of SARS-CoV-2 transcriptome exhibited that the percentage of viral data approached 70% (Kim et al., 2020). Therefore, without exogenous contamination, the dramatically transcriptional differences between the two PRRSV strains may be attributed to the discrepancy in the viral transcriptional capacity. In addition, three hub genes were selected from the mRNA co-expression network constructed by WGCNA. Among them, *GLMP* is required to protect lysosomal transporter *MFSD1* from lysosomal proteolysis and is often involved in protein localization to the lysosome (López et al., 2019). Research has found that *LAPTM4A* functions to regulate the compartmentalization of amphiphilic solutes within lysosomes and late endosomes (Vergarajauregui et al., 2011). *LITAF*, as the small integral membrane protein of lysosome/endosome, plays a part in proteins’ lysosomal degradation and the expression regulation of various cytokines (such as *TNF, CCL2, CCL5, IL1A*) (Myokai et al., 1999; Tang et al., 2005). In this study, PRRSV propagation was significantly inhibited in both mRNA and protein levels after overexpressing three genes. As discussed above, we speculate that the inhibition may cause by disruption of lysosome structure/function, but the mechanism still needs further exploration.

## Data availability statement

The datasets presented in this study can be found in online repositories. The names of the repository/repositories and accession number(s) can be found at: https://www.ncbi.nlm.nih.gov/, PRJNA857481.

## Ethics statement

All the animal-related experiments were handled according to the Ethics Committee at Northwest A&F University (approval number DY2022009). Experiments were carried out following the approved guidelines.

## Author contributions

XLW and PXW designed the experiments, and PXW conducted the bioinformatics analyses; XM, YXZ, and RCH performed the data validation; XLW, PXW, RTZ, CL, and BZ wrote the manuscript; ZQY, JW, HJL and LQ guided the research. All authors read and approved the final manuscript.

## Funding

This research was supported by grants from the National Natural Science Foundation of China awarded to XW (grant no. 31672581) and the grants from the General Projects of Key R&D Program in Shaanxi Province (2022SF-421).

## Conflict of interest

The authors declare that the research was conducted in the absence of any commercial or financial relationships that could be construed as a potential conflict of interest.

## Publisher’s note

All claims expressed in this article are solely those of the authors and do not necessarily represent those of their affiliated organizations, or those of the publisher, the editors and the reviewers. Any product that may be evaluated in this article, or claim that may be made by its manufacturer, is not guaranteed or endorsed by the publisher.

## Supplementary material

SUPPLEMENTARY FIGURE 1 Diagram of the experimental design. The sample preparation process used for RNA-Seq in this study;

SUPPLEMENTARY FIGURE 2 DEGs analysis between JXA1 and NC group. **(A)** Volcano plots show the dynamic change of DEGs under each comparison. Each gene’s change state is mapped by color, and the horizontal and vertical dash lines represent the DEGs cutoff (LFC = ±1, padj = 0.05). **(B)** Heat map of the expression of the top 20 up- and down-regulated DEGs (sorted by LFCs) at three timepoints.

SUPPLEMENTARY FIGURE 3 Functional annotation analysis of DEGs determined from the JXA1 and NC group. **(A)** GO enrichment at different hpi. The left side of the panel shows the significantly enriched GO terms (|NES| ≥ 1.5, padj < 0.1) obtained at all three hpi. Gene sets are represented by nodes, which color corresponds to hpi; node size corresponds to the number of gene members within them; the size and color of the edges correspond to the similarity score between terms. All GO terms are clustered according to their gene members’ overlap size, and each cluster’s subject is written on top. GO terms associated with immune response, antigen processing and presentation are shown in the panel’s lower and upper bar plots on the right side. Each bar contains a padj of the permutation test. **(B)** Lollipop plot of the significantly enriched KEGG pathway at different hpi. **(C)** Heat map of the expression profiles of hotspot genes associated with innate immunity. Each layer of circles represents a comparison group, and the selected genes include common cytokines, ISGs, MHCs, and critical regulators in associated pathways.
