# Supplementary figures and images for: The transcriptional characteristics of NADC34-like PRRSV in porcine alveolar macrophages
Source: Front Microbiol. 2022 Oct 19;13:1022481. doi: 10.3389/fmicb.2022.1022481 (PMC9629508; doi:10.3389/fmicb.2022.1022481)

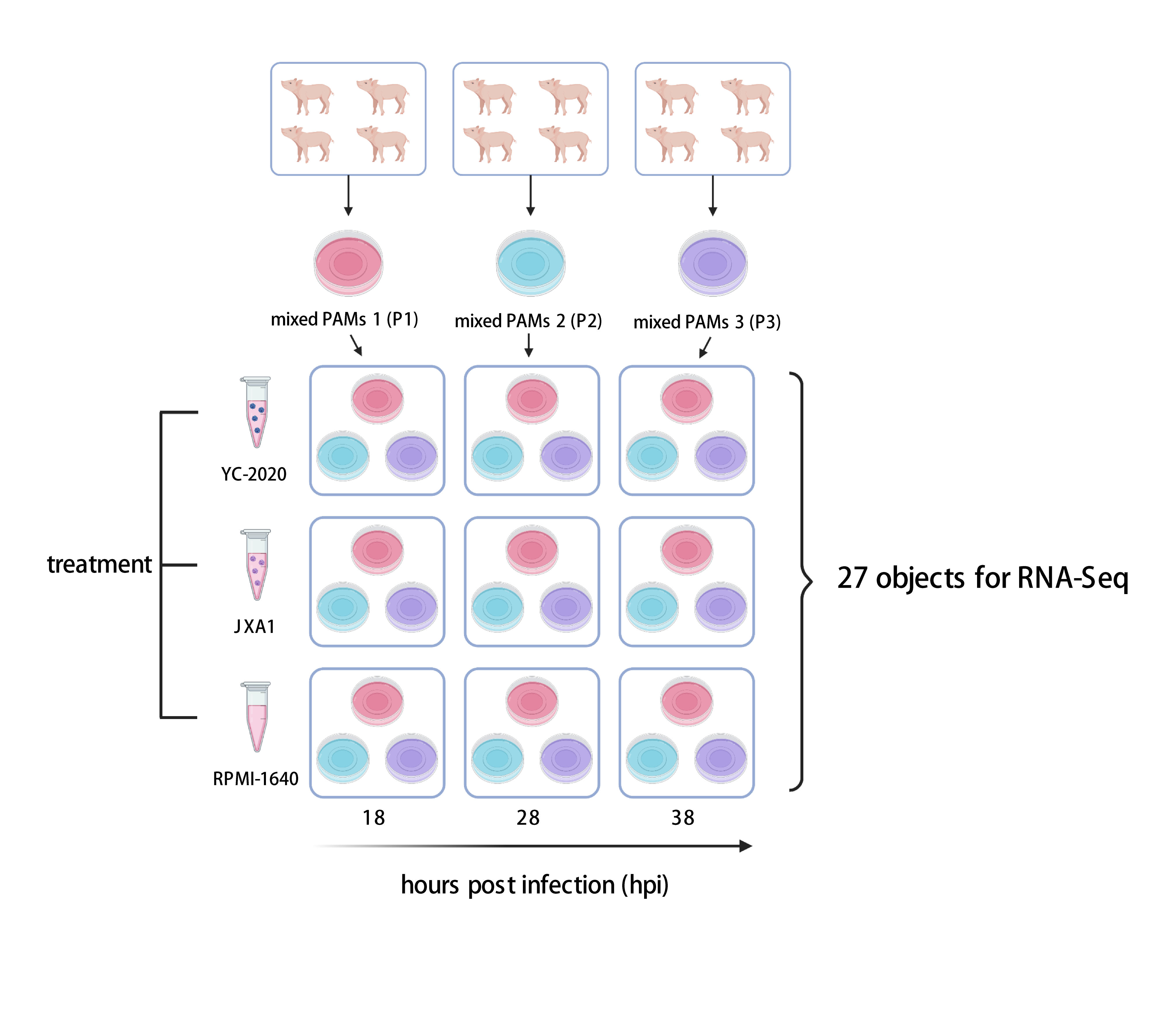

Supplement: SUPPLEMENTARY FIGURE 1 — Diagram of the experimental design. The sample preparation process used for RNA-Seq in this study; [file Image_1.TIF]

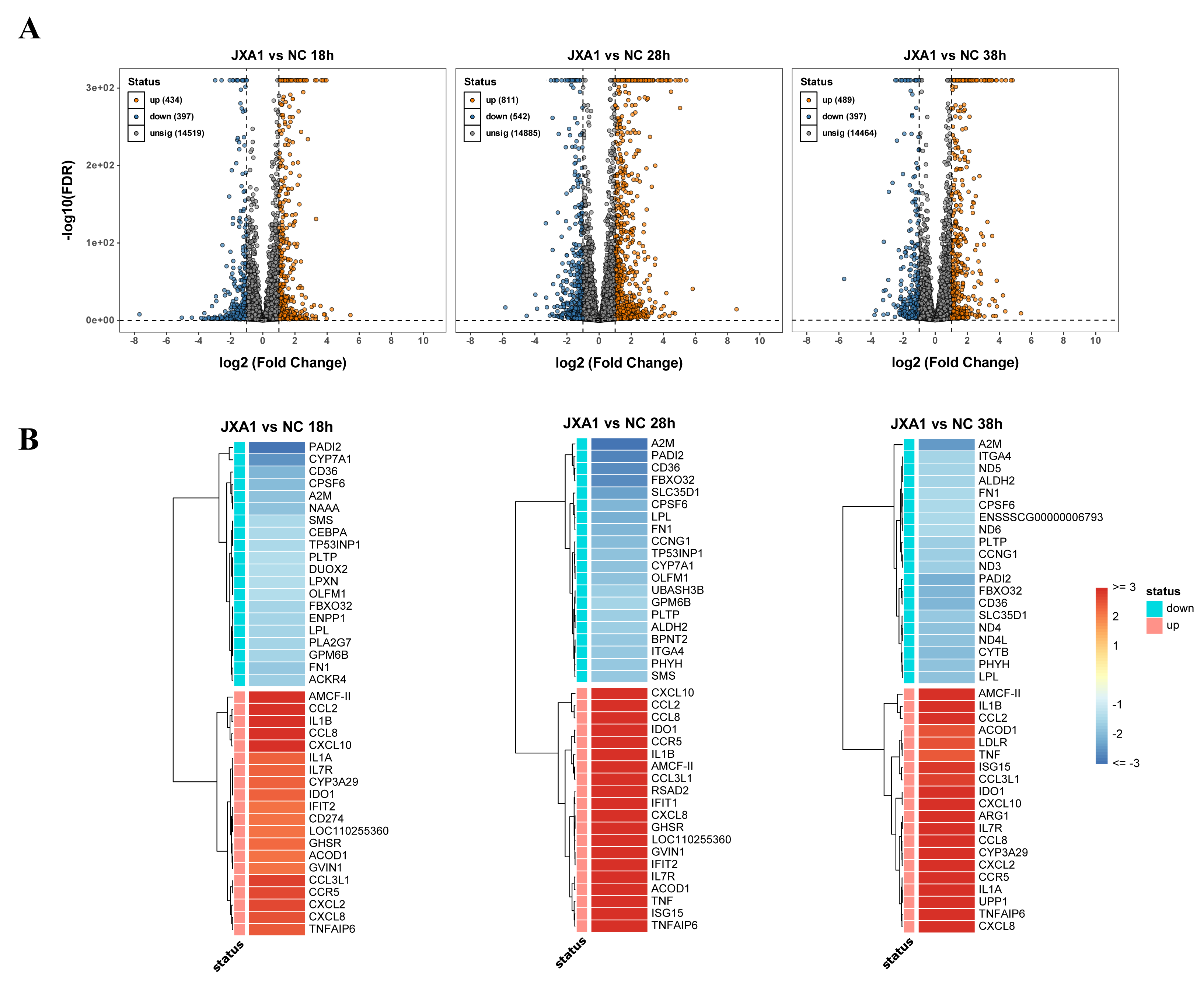

Supplement: SUPPLEMENTARY FIGURE 2 — DEGs analysis between JXA1 and NC group. (A) Volcano plots show the dynamic change of DEGs under each comparison. Each gene’s change state is mapped by color, and the horizontal and vertical dash lines represent the DEGs cutoff (LFC = ±1, padj = 0.05). (B) Heat map of the expression of the top 20 up- and down-regulated DEGs (sorted by LFCs) at three timepoints. [file Image_2.TIF]

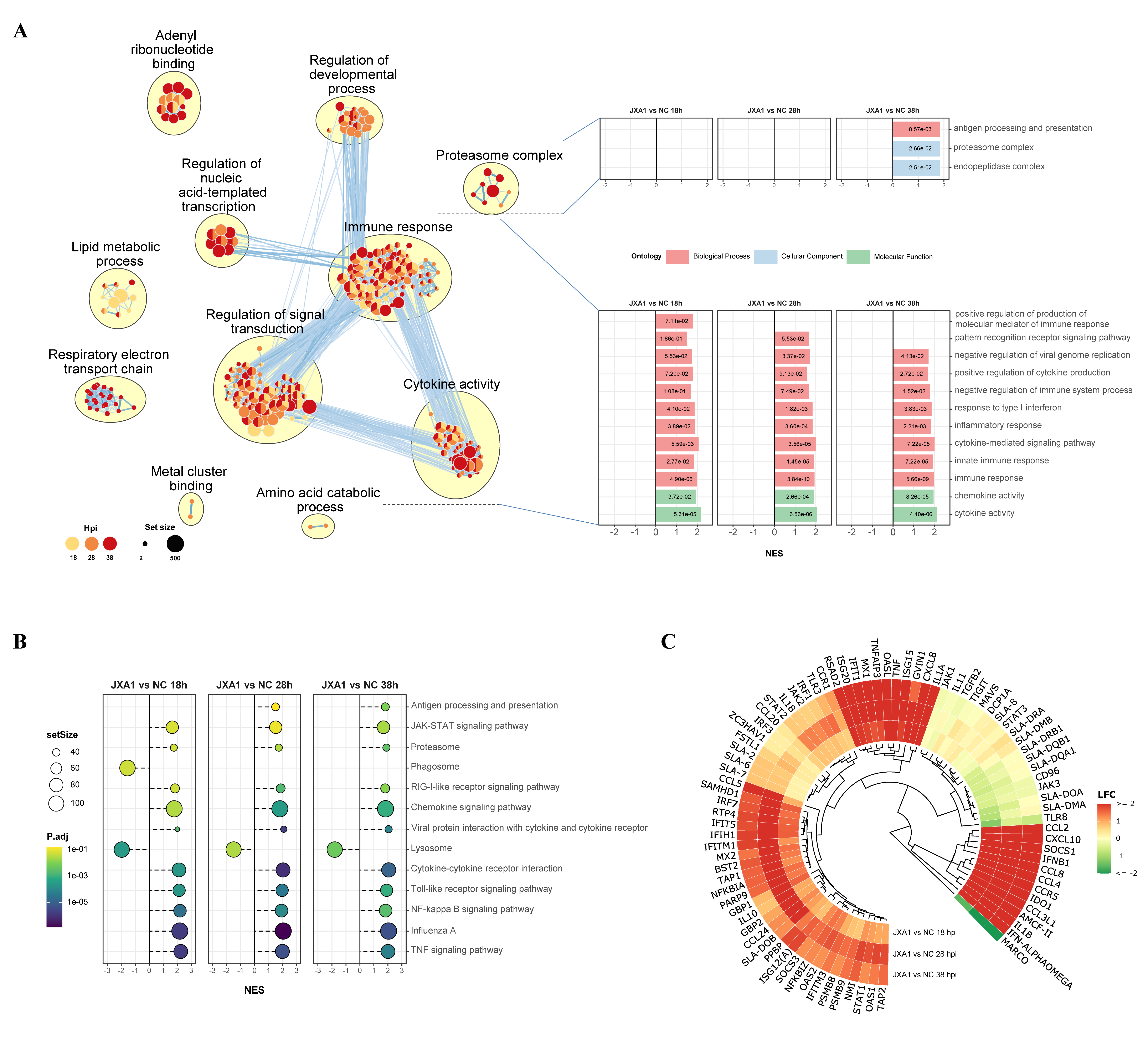

Supplement: SUPPLEMENTARY FIGURE 3 — Functional annotation analysis of DEGs determined from the JXA1 and NC group. (A) GO enrichment at different hpi. The left side of the panel shows the significantly enriched GO terms (|NES| ≥ 1.5, padj < 0.1) obtained at all three hpi. Gene sets are represented by nodes, which color corresponds to hpi; node size corresponds to the number of gene members within them; the size and color of the edges correspond to the similarity score between terms. All GO terms are clustered according to their gene members’ overlap size, and each cluster’s subject is written on top. GO terms associated with immune response, antigen processing and presentation are shown in the panel’s lower and upper bar plots on the right side. Each bar contains a padj of the permutation test. (B) Lollipop plot of the significantly enriched KEGG pathway at different hpi. (C) Heat map of the expression profiles of hotspot genes associated with innate immunity. Each layer of circles represents a comparison group, and the selected genes include common cytokines, ISGs, MHCs, and critical regulators in associated pathways. [file Image_3.TIF]
